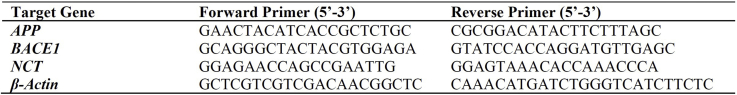# Degradation and inhibition of epigenetic regulatory protein BRD4 exacerbate Alzheimer’s disease-related neuropathology in cell models

**DOI:** 10.1016/j.jbc.2022.101794

**Published:** 2022-03-03

**Authors:** Siyi Zhang, Ping Bai, Dan Lei, Yingxia Liang, Sherri Zhen, Grisilda Bakiasi, Hao Pang, Se Hoon Choi, Changning Wang, Rudolph E. Tanzi, Can Zhang

**Affiliations:** 1 Genetics and Aging Research Unit, McCance Center for Brain Health, MassGeneral Institute for Neurodegenerative Diseases (MIND), Department of Neurology, Massachusetts General Hospital and Harvard Medical School, Charlestown, Massachusetts, USA; 2 Department of Forensic Medicine, China Medical University, Shenyang, China; 3 Athinoula A. Martinos Center for Biomedical Imaging, Department of Radiology, Massachusetts General Hospital and Harvard Medical School, Charlestown, Massachusetts, USA

**Keywords:** Alzheimer’s disease, amyloid-β, tau, amyloid-β protein precursor, epigenetics, bromodomain-containing protein 4, BACE1, nicastrin, amyloidogenesis, AD, Alzheimer’s disease, Aβ, amyloid-β, APP, amyloid-β protein precursor, BRD4, bromodomain-containing protein 4, BET, bromodomain and extra-terminal protein, EHMT2, euchromatic histone-lysine N-methyltransferase 2, NCT, nicastrin, P-TEFb, positive transcriptional elongation factor b, PROTAC, proteolysis targeting chimeras, p-Tau, pTau, phosphorylated Tau, t-Tau, total Tau, WB, Western blotting, Q-PCR, quantitative polymerase chain reaction

## Abstract

Epigenetic regulation plays substantial roles in human pathophysiology, which provides opportunities for intervention in human disorders through the targeting of epigenetic pathways. Recently, emerging evidence from preclinical studies suggested the potential in developing therapeutics of Alzheimer’s disease (AD) by targeting bromodomain containing protein 4 (BRD4), an epigenetic regulatory protein. However, further characterization of AD-related pathological events is urgently required. Here, we investigated the effects of pharmacological degradation or inhibition of BRD4 on AD cell models. Interestingly, we found that both degradation and inhibition of BRD4 by ARV-825 and JQ1, respectively, robustly increased the levels of amyloid-beta (Aβ), which has been associated with the neuropathology of AD. Subsequently, we characterized the mechanisms by which downregulation of BRD4 increases Aβ levels. We found that both degradation and inhibition of BRD4 increased the levels of BACE1, the enzyme responsible for cleavage of the amyloid-beta protein precursor (APP) to generate Aβ. Consistent with Aβ increase, we also found that downregulation of BRD4 increased AD-related phosphorylated Tau (pTau) protein in our 3D-AD human neural cell culture model. Therefore, our results suggest that downregulation of BRD4 would not be a viable strategy for AD intervention. Collectively, our study not only shows that BRD4 is a novel epigenetic component that regulates BACE1 and Aβ levels, but also provides novel and translational insights into the targeting of BRD4 for potential clinical applications.

Alzheimer’s disease (AD) is the most common cause of dementia, which tremendously causes worldwide financial loss and leads to severe social burden (1, 2). Because of the urgency of this situation, a better understanding of AD etiology and effective treatments requires to be discovered. The neuropathology of AD has been well characterized by two hallmarks in AD brains. They include the extracellular amyloid plaques, which are primarily formed by deposition of the β-amyloid (Aβ) peptides, which is usually early pathological manifestations of AD, and the intraneuronal neurofibrillary tangles within the dystrophic neurites, which consist of the aggregated and pathologic Tau proteins, and usually occurs in later stages of AD following Aβ accumulation (3). Although the pathogenesis of AD is complex and has not been completely elucidated, investigation of disease neuropathology has led to considerable evidence supporting “*Aβ hypothesis.*” It posits that Aβ upregulation and accumulation are the primary pathological events of AD, which induce phosphorylated Tau (pTau) in neurons, followed by neurofibrillary tangle formation and neuroinflammation, as well as synaptic dysfunction and loss, and ultimately, neurodegeneration (4, 5, 6, 7).

Recently, there has been keen interest focusing on the functions of epigenetics underlying AD pathogenesis as well as targeting epigenetics for developing therapeutics of AD (8, 9). Epigenetic factors regulate gene transcription and respond to environmental changes and show widespread remodeling during aging (8). The pathogenesis for AD involves important and complex epigenetic mechanisms (10, 11). Therefore, identification and elucidation of molecular mechanisms by which epigenetic factors contribute to AD pathological changes may provide novel insights of AD etiology and generate new strategies with therapeutic potentials for AD treatment.

Bromodomain-containing protein 4 (BRD4) is a recent focus of epigenetic regulation research, which has been evaluated for both biological functions and clinical potentials. As a member that belongs to the bromodomain and extraterminal (BET) protein family, BRD4 acts as an epigenetic regulator with multiple roles in gene transcription regulation (12, 13, 14, 15). First, it binds to the acetylated histones at the enhancers, promoters, and transcription start sites though its acetyl-lysine recognition pocket to enhance gene transcription. BRD4 is well characterized for a positive regulating mechanism involving positive transcriptional elongation factor b (P-TEFb) (13, 14, 15, 16). It also associates with the histone acetyltransferases P300 and CBP to enhance H3K27 acetylation (12). These properties make BRD4 an important component of a superenhancer (16). In addition to its role as a positive regulator, BRD4 is a transcriptional suppressor of several autophagy-related genes by interacting with the euchromatic histone-lysine N-methyltransferase 2 (EHMT2) (17, 18), which negatively regulates autophagy activity. Biologically, BRD4 is crucial to cell growth; mutations in *BRD4* are related to several abnormalities, including altered differentiation and apoptosis on cellular levels (13). Individuals carrying mutations in *BRD4* show neuroskeletal abnormalities (19, 20, 21). Additionally, BRD4 is critical to neuronal function and mediates transcriptional regulation related to learning and memory (22).

Because BRD4 is a key player that affects human pathophysiology, it has become an increasingly important target for clinical therapeutic development. Many compounds such as BRD4-related inhibitors and proteolysis targeting chimeras (PROTAC) have been developed and evaluated in clinical trials (23, 24, 25, 26, 27). Some compounds under clinical trials were included in Table S1. In addition to anticancer applications, BRD4 has been suggested to be a valid target in type-I diabetes and heart failure from preclinical studies (25, 26). Additionally, preclinical evidence suggested that BRD4 can be a potential target for AD intervention. JQ1 is a well-studied small molecule that is a prototype inhibitor of BET proteins (28, 29). It has been evaluated on the pathological and behavioral levels. On the pathological levels, JQ1 reduces pTau (Ser396) levels in the 3× transgenic AD mice expressing APP/PS1/Tau (30). JQ1 also regulates microglia and inflammatory responses (31, 32, 33). In wild-type animals, it improves brain plasticity with positive effects on cognitive performance (34), but shows negative effects on neuronal activity and AD-related behavorial studies in a different study (22). In APP/PS1 transgenic mice, JQ1 rescues cognitive deficits (34). These findings suggest that BRD4 protein plays complex and key functions in pathophysiology of AD. Presently, the effects of BRD4 on Aβ generation remain largely unknown and require further characterization.

Here, our study has been designed to focus on the effects of pharmacological regulation of BRD4 on Aβ-overexpressing AD cell models, using both degradation and inhibition mechanisms. We envision that our study will provide novel and translational insights toward BRD proteins in clinical applications related to AD.

## Results

### Regulation of ARV-825 and JQ1 on Aβ levels and APP processing in H4-APP751 cells

**Supplemental Figure S1:**
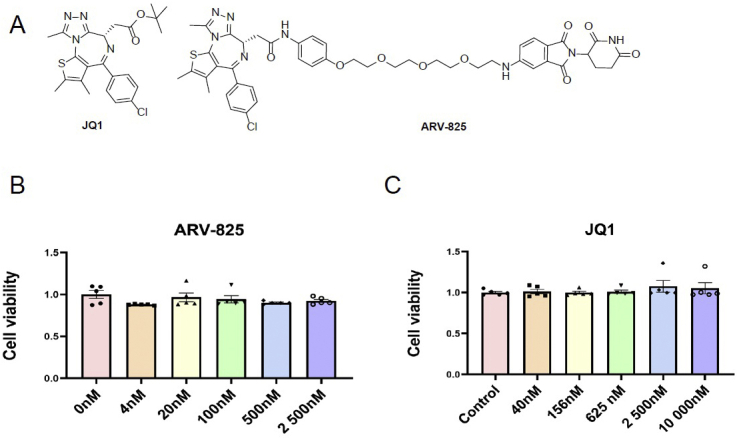


In this study, we first set out to determine the molecules and approaches to enable pharmacological regulation of BRD4. Recently, increasing numbers of BET modulators have been developed and many compounds have entered clinical trials. We searched online for related clinical trials (https://clinicaltrials.gov/), which led to identification of dozens of trials for BET inhibitors (Table S1). We chose two prototype molecules that either degrade or inhibit BET/BRD proteins, particularly BRD4. They included ARV-825, a proteolysis-targeting chimera (PROTAC)-based molecule for degrading (35) and JQ1 for inhibiting BET/BRD (28), respectively. ARV-825 is structurally related to OTX015, a BET inhibitor currently in a Phase-II trial (Fig. S1*A*). JQ1 has been extensively investigated in preclinical studies and provides an effective and classic tool to study the role of BRD4, although it was not applied to clinical trial due to its very short half-life (36).

**Figure 1:**
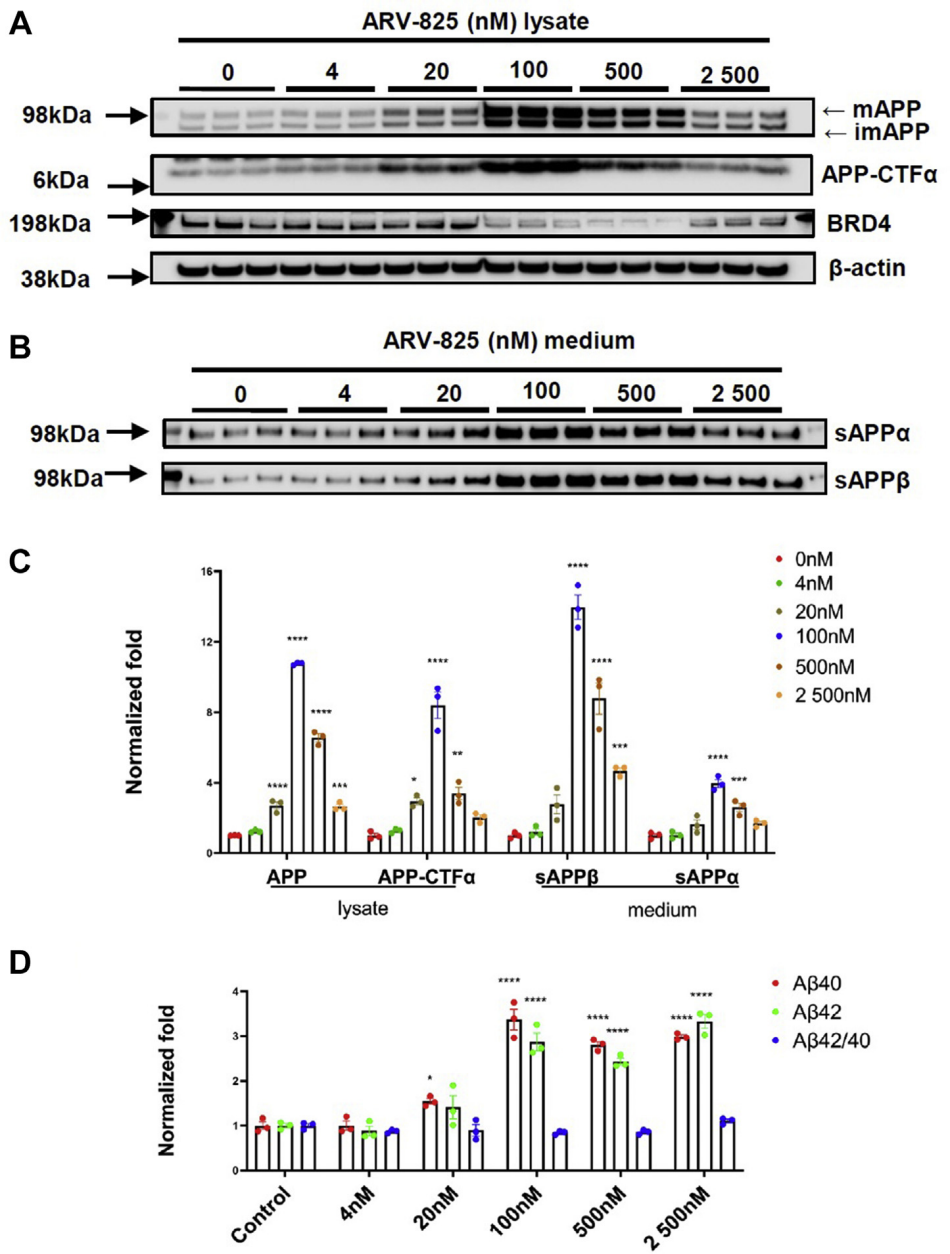
**Degradation of BRD4 by ARV-825 upregulates Aβ levels and APP processing in H4-APP751 cells.** Cells were treated with ARV-825 (nM; 4, 20, 100, 500 and 2500) for 18 h. Conditioned medium was applied to MSD-Aβ analysis to assess Aβ levels. Cell lysates and conditioned medium were applied to WB to detect BRD4 and APP processing pathway. Lysates were probed with G12A (for APP-FL/CTF) and β-actin (as a control protein) and medium was probed with antibodies for sAPPα (by 6E10) and sAPPβ, respectively. *A*–*C*, WB analysis of BRD4 and APP processing pathway. *D*, MSD-Aβ analysis for Aβ levels. Mean ± SEM; n = 3; one-way ANOVA followed by Dunnett’s test; ∗/∗∗/∗∗∗/∗∗∗∗ showed significance; ∗*p* < 0.05; ∗∗*p* < 0.01; ∗∗∗*p* < 0.001; ∗∗∗∗*p* < 0.0001. Aβ, amyloid-β; APP, amyloid-β protein precursor; BRD4, bromodomain-containing protein 4.

For our cell-based studies, we investigated the effects of ARV-825 on Aβ levels and APP processing in the human neuroblastoma H4-APP751 model, a previously reported cell model stably overexpressing APP. Cells were treated with ARV-825 of different doses (nM; 4, 20, 100, 500, and 2500) for 18 h. Conditioned medium was applied to MSD-Aβ analysis to assess Aβ levels. Cell lysates and conditioned medium were used to WB to detect APP processing pathway. We found that the cells displayed good tolerance and no floating cells were observed under the microscopy. Cell viabilities were also tested by alamarBlue test. Our results showed that there were no significant differences among the treatment groups of various doses (Fig. S1*B*). We performed WB analysis in cell lysates to assess APP processing and BRD4 protein levels (Fig. 1, *A*–*C*) and carried out MSD-Aβ analysis in conditioned medium to assess Aβ levels. We showed that ARV-825 reduced BRD4 protein levels, as expected and due to PROTAC-related BRD4 degradation (Fig. 1, *A* and *B*). This also agreed with previous findings (35). Interestingly, we found that Aβ levels were significantly increased as a function of ARV-825 (*p* < 0.05) (Fig. 1*C*). We also showed that ARV-825 did not significantly change Aβ(42:40) ratios (*p* > 0.05) (Fig. 1*D*).

Furthermore, we asked if the effects of ARV-825 on Aβ increase were related to APP processing. We showed that ARV-825 displayed robust effects on regulating the levels of APP full-length and APP-CTF in lysates and levels of sAPPα and sAPPβ in conditioned medium. The APP-CTFβ levels were not able to be detected. The changes of Aβ, APP-CTFα, and sAPPα levels were consistent with APP-FL (Fig. 1, *A*–*C*). Among these proteins, the upregulation of sAPPβ displayed highest level of change, up to 14.3-fold increase *versus.* control (Fig. 1, *B* and *C*). Compared with the control, highest levels of increase of these proteins were associated with 100 nM dose (*p* < 0.05, APP-FL, to 1097.7% ± 21.6%; sAPPα, to 396.0% ± 39.8%; sAPPβ, to 1434.1% ± 156.5%; APP-CTFα, to 864.4% ± 148.3%; Aβ40–337.2% ± 40.0%, Aβ42–287.5% ± 33.5%) (Fig. 1, *C* and *D*). Collectively, ARV-825 is a key player that significantly upregulates Aβ levels by increasing APP-FL levels and regulating APP processing in H4-APP751 cells.

**Figure 2:**
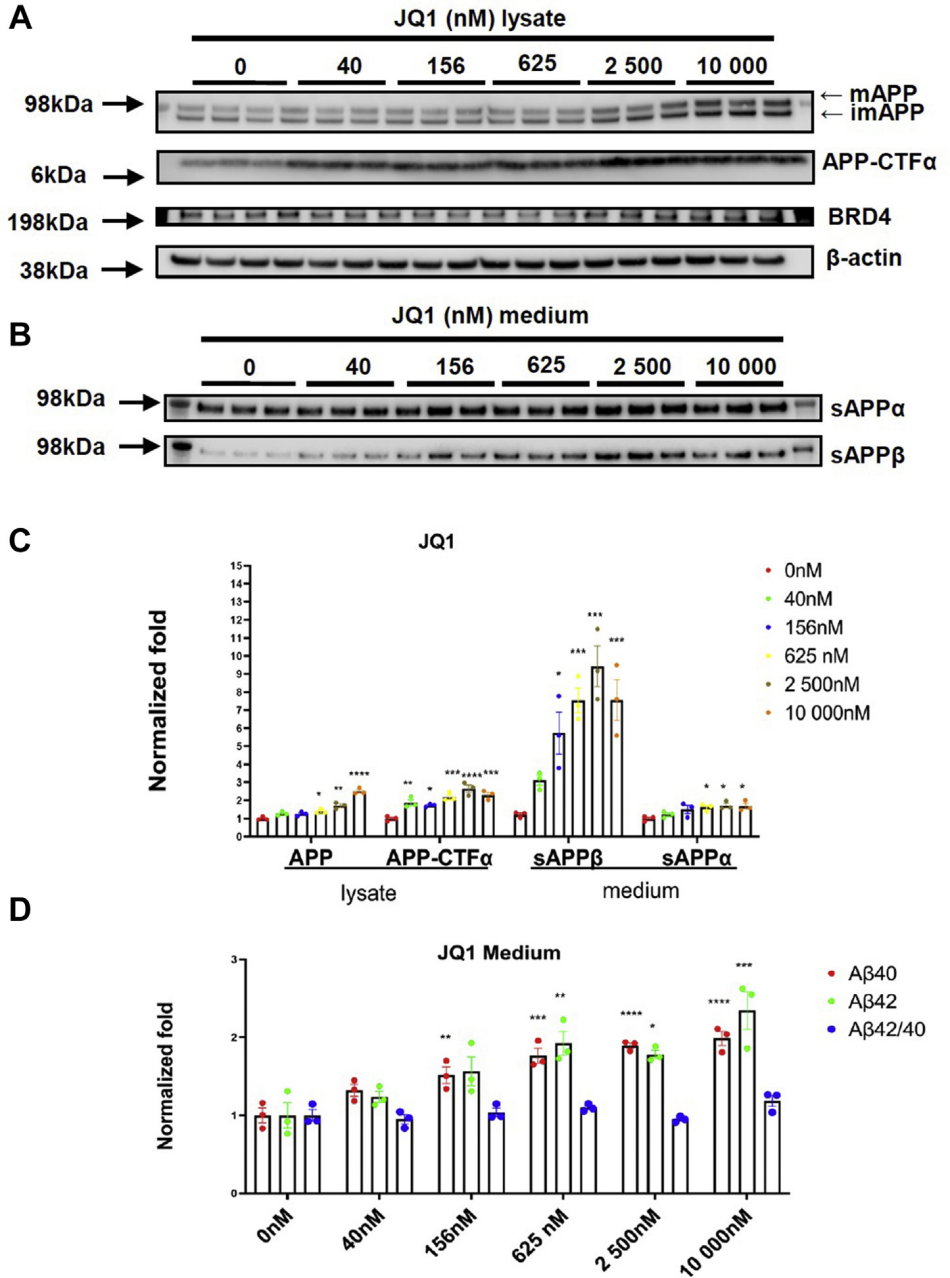
**Inhibition of BRD4 by JQ1, upregulates Aβ levels and APP processing in H4-APP751 cells.** Cells were treated with JQ1 (nM; 40, 160, 625, 2500 and 10,000) for 18 h. Conditioned medium was applied to MSD-Aβ analysis to assess Aβ levels. Cell lysates and conditioned medium were applied to WB to detect BRD4 and APP processing pathway. Lysates were probed with antibodies of BRD4, G12A (for APP-FL/CTF) and β-actin. Medium was probed with antibodies for sAPPα (by 6E10) and sAPPβ, respectively. *A*–*C*, WB analysis of BRD4 and APP processing pathway after JQ1 treatment. *D*, MSD-Aβ analysis for Aβ levels after JQ1 treatment. Mean ± SEM; n = 3; one-way ANOVA followed by Dunnett’s test; ∗/∗∗/∗∗∗/∗∗∗∗ showed significance; ∗*p* < 0.05; ∗∗*p* < 0.01; ∗∗∗*p* < 0.001; ∗∗∗∗*p* < 0.0001. Aβ, amyloid-β; APP, amyloid-β protein precursor; BRD4, bromodomain-containing protein 4.

Next, we analyzed if JQ1-mediated BRD4 inhibition may also change Aβ levels and APP processing. H4-APP751 cells were treated with various concentrations of JQ1 (nM; 40, 160, 625, 2500, and 10,000) for 18 h. Cell viability analysis by the alamarBlue study showed no significant differences from different groups (Fig. S1*C*). Then conditioned medium was applied to MSD-Aβ analysis to assess Aβ levels; cell lysates and conditioned medium were applied by WB to detect APP processing. We found that JQ1 dose-dependently increased Aβ levels (*p* < 0.05) without significantly affecting Aβ(42:40) ratios (Fig. 2*D*). Additionally, JQ1 dose-dependently increased levels of APP-FL, APP-CTF, sAPPα, and sAPPβ (Fig. 2, *B*–*D*). For example, at 625 nM, JQ1 led to a robust increase in the levels of sAPPα/β (both *p* values <0.05; sAPPα, to 189.3% ± 35.1%; sAPPβ, to 367.9% ± 85.8%), APP-FL (*p* < 0.05, to 152.4% ± 14.0%), and APP-CTFα (*p* < 0.05, to 180.5% ± 26.9%). Thus, both ARV-825 and JQ1 upregulates Aβ levels by increasing APP-FL levels and regulating APP processing in H4-APP751 cells.

### Upregulation of BACE1 levels by ARV-825 and JQ1 in H4-APP751 cells

**Figure 3:**
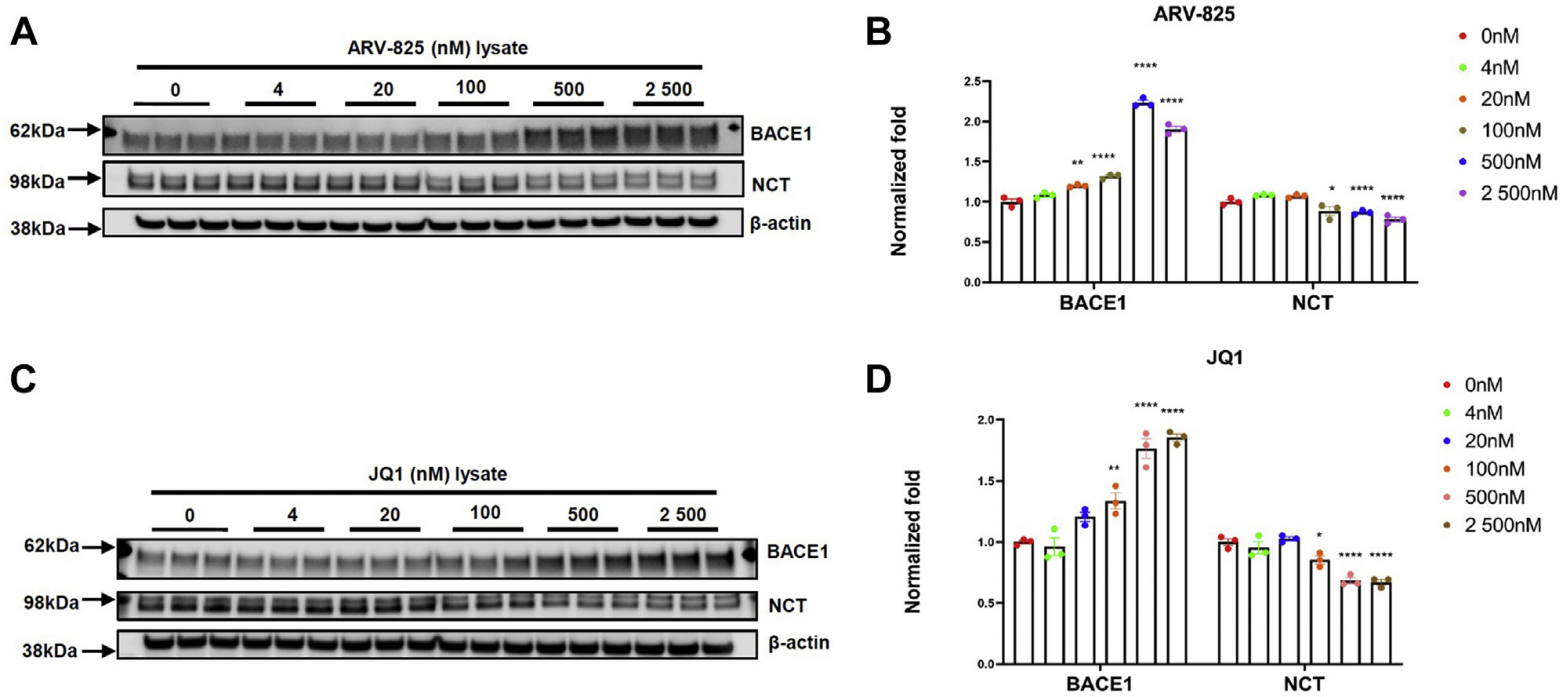
**ARV-825 and JQ1 upregulate BACE1 levels in H4-APP751 cells.** Cells were treated with ARV-825 (nM; 4, 20, 100, 500, and 2500) or JQ1 (nM; 4, 20, 100, 500, and 2500) for 18 h. Cell lysates were collected and applied to WB to detect BACE1 and NCT levels, with β-actin utilized as a control protein. *A* and *B*, WB analysis for effects of ARV-825. *C* and *D*, WB analysis for effects of JQ1. Mean ± SEM; n = 3; one-way ANOVA followed by Dunnett’s test; ∗/∗∗/∗∗∗/∗∗∗∗ showed significance; ∗*p* < 0.05; ∗∗*p* < 0.01; ∗∗∗*p* < 0.001; ∗∗∗∗, *p* < 0.0001. NCT, nicastrin.

**Supplemental Figure S2:**
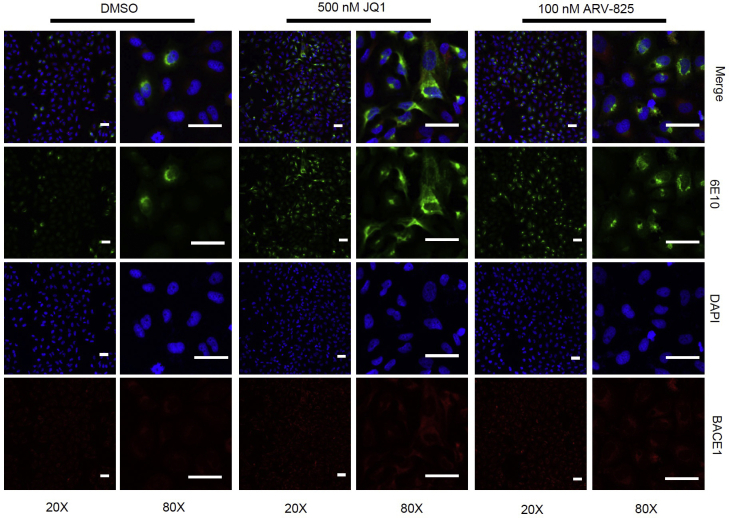


Upregulation of Aβ generation can be caused by mechanisms involving β-/γ-secretase-related APP processing or APP levels. We, next, asked if these compounds change BACE1 protein levels. H4-APP751 cells were treated with ARV-825 or JQ1 (nM; 4, 20, 100, 500 and 2500) and harvested. Cell lysates were applied to WB and probed with the BACE1 antibody. We showed that BACE1 levels were significantly upregulated by both ARV-825 and JQ1(Fig. 3). In total, 100 nM ARV-825 and 500 nM JQ1 increased BACE1 levels up to 1.37-fold and 1.76-fold, respectively, which supported Aβ increases at these doses (Fig. 3). In addition to WB, we utilized the confocal immunofluorescence (IF) microscopy analysis for cellular expression of BACE1 and APP. H4-APP751 cells were treated with vehicle, 100 nM ARV-825 or 500 nM JQ1 for 18 h and applied to IF analysis. We showed that the expression patterns of APP and BACE1 from the cells treated with ARV-825 or JQ1 were comparable to control-treated cells (Fig. S2). Collectively, the mechanism of both ARV-825 and JQ1 induced Aβ increase in H4-APP751 cells was associated with the increase of BACE1 levels.

### Effects of ARV-825 and JQ1 on NCT levels in H4-APP751 cells

**Supplemental Figure S3:**
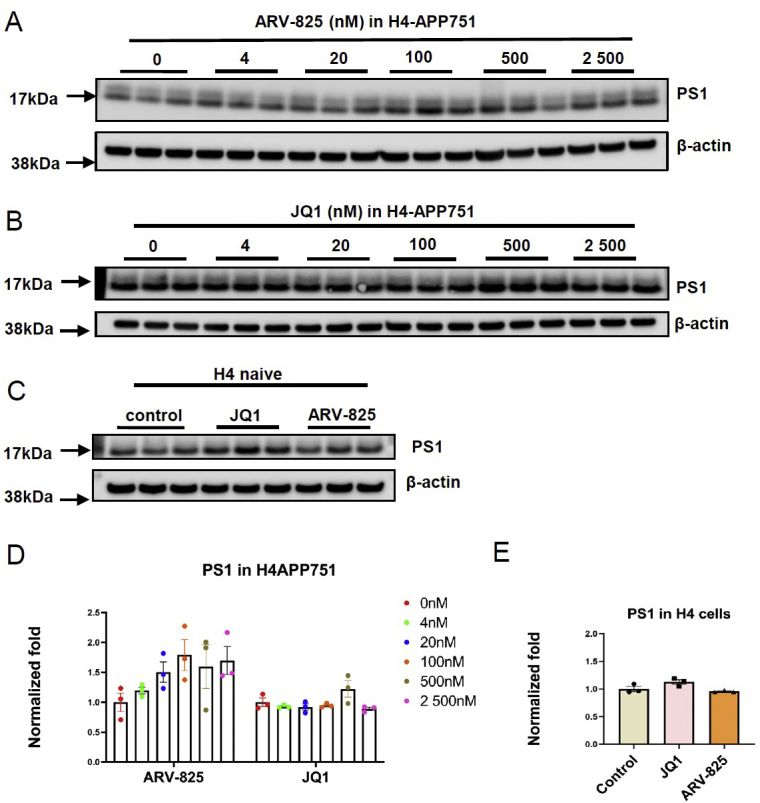


So far, we showed that ARV-825 and JQ1 upregulated Aβ levels through mechanisms involving increases of BACE1 and APP levels. Four components of γ-secretase cross-regulate each other; downregulation or deficiency of one component may destabilize and affect γ-secretase (37). Among the four components, PS1 harbors the catalytic activity of γ-secretase and closely relates to AD. PS1 changes, particularly those in familial AD (FAD) mutations, usually lead to changes in Aβ(42:40) ratios (38, 39, 40). A previous study using JQ1-treated animals showed that PS1 mRNA expression was not significantly affected (34). - Next, we investigated the effects of ARV-825 or JQ1 on γ-secretase component levels by WB analysis. Using an antibody for the PS1-CTF, we showed that ARV-825 or JQ1 did not significantly change PS1-CTF levels comparing with control (Fig. S3, *A* and *B*). Next, we studied the effects of ARV-825 or JQ1 on nicastrin (NCT), a γ-secretase component, which is essential for γ-secretase assembly and function (41). Interestingly, in H4-APP751 cells, ARV-825 downregulated NCT levels; at the dose of 500 nM, ARV-825 downregulated NCT levels to 87.4% *versus.* control (*p* < 0.05) (Fig. 3, *A* and *B*). Additionally, JQ1 dose-dependently reduced NCT levels: 100 nM JQ1 decreased NCT levels down to 85.4% *versus.* control (*p* < 0.05), and 500 nM JQ1 decreased NCT levels down to 68.4% *versus.* control (*p* < 0.05) (Fig. 3, *C* and *D*). Thus, ARV-825 or JQ1 increased Aβ levels by mechanisms primarily involving an upregulation of BACE1 levels and β-secretase-mediated APP processing, but not by increasing γ-secretase levels and activity.

### Analysis of BACE1 protein and mRNA levels by ARV-825 and JQ1 in naïve H4 cells

**Figure 4:**
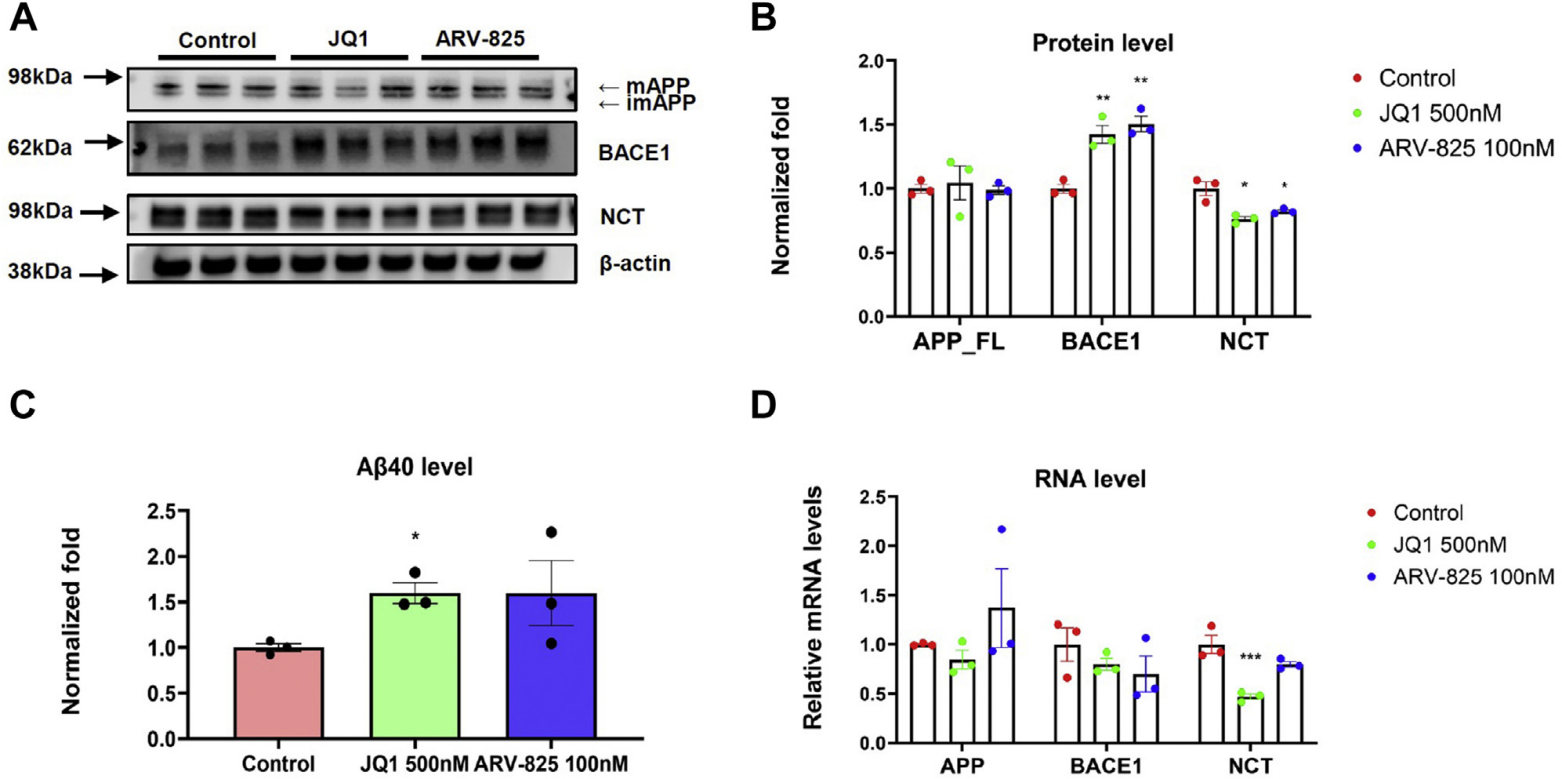
**Analysis of APP, BACE1 and NCT levels by ARV-825 or JQ1 in naïve H4 cells.** Cells were treated with ARV-825 100 nM, JQ1 500 nM and DMSO (control) for 18 h, followed by WB (*A* and *B*), MSD-Aβ (*C*), and RT-qPCR (*D*) analysis. *A*, protein analysis by WB. Cell lysates were probed with antibodies of BACE1, NCT, and G12A (for APP) and β-actin (as a control protein). *B*, quantification of *A*. *C*, MSD-Aβ analysis for Aβ levels. Aβ40 proteins were detected and analyzed. Aβ42 proteins were not able to be detected because of low concentrations. *D*, RT-qPCR analysis of mRNA levels for *APP*, *BACE1* and *NCT* by JQ1 or ARV-825 in naïve H4 cells. Mean ± SEM; n = 3; one-way ANOVA followed by Dunnett’s test; ∗/∗∗/∗∗∗ showed significance; ∗*p* < 0.05; ∗∗*p* < 0.01; ∗∗∗*p* < 0.001. APP, amyloid-β protein precursor; NCT, nicastrin.

Next, because AD-related Aβ may result in BACE1 increase (42), we tested whether BACE1 upregulation by ARV-825 and JQ1 may also occur in naïve cells. Particularly, we utilized H4 naïve cells, which endogenously express low Aβ levels. The H4 naïve cells were treated for 18 h with 100 nM ARV-825 and 500 nM JQ1, the doses of which recapitulated those that displayed strong effects on regulating Aβ levels and APP processing in H4-APP751 cells. The cell lysates and medium were respectively applied to WB (Fig. 4, *A* and *B*) and MSD-Aβ analysis (Fig. 4*C*). Notably, we found that ARV-825 or JQ1 significantly increased BACE1 levels, respectively, compared with control (*p* < 0.05) (Fig. 4, *A* and *B*). Furthermore, ARV-825 or JQ1 decreased NCT levels, respectively, compared with control (*p* < 0.05). We also studied the effects of ARV-825 or JQ1 on APP-FL levels. We found no significant changes on APP-FL levels comparing ARV-825 or JQ1 with control in naïve H4 cells (Fig. 4, *A* and *B*). We performed MSD-Aβ analysis and found that Aβ42 proteins were not able to be detected. We showed that a significant upregulation of Aβ40 levels was observed in association with JQ1 (*p* < 0.05) and a trend toward an increase of Aβ40 levels as a function of ARV-825 (*p* > 0.05) (Fig. 4*C*).

We next performed the RT-qPCR analysis to investigate whether downregulation of BRD4-related BACE1 increase by ARV-825 or JQ1 may occur through a transcriptional or a posttranscriptional mechanism. H4 naïve cells were treated with 100 nM ARV-825, 500 nM JQ1, or DMSO for 18 h and applied to RT-qPCR analysis for *BACE1* mRNA levels. We found no significant differences in *BACE1* mRNA levels comparing respectively the ARV-825 or JQ1 group with the control group (*p* > 0.05) (Fig. 4*D*). These results suggested that BRD4-related regulation of BACE1 was using a posttranscriptional mechanism. Furthermore, RT-qPCR analysis of *NCT* mRNA levels in JQ1 group showed a reduction of *NCT* down to 47.0% ± 5.0% in naïve H4 cells (*p* < 0.05) (Fig. 4*D*).

### Analysis of tau changes in 3D-AD human neural cell culture

**Figure 5:**
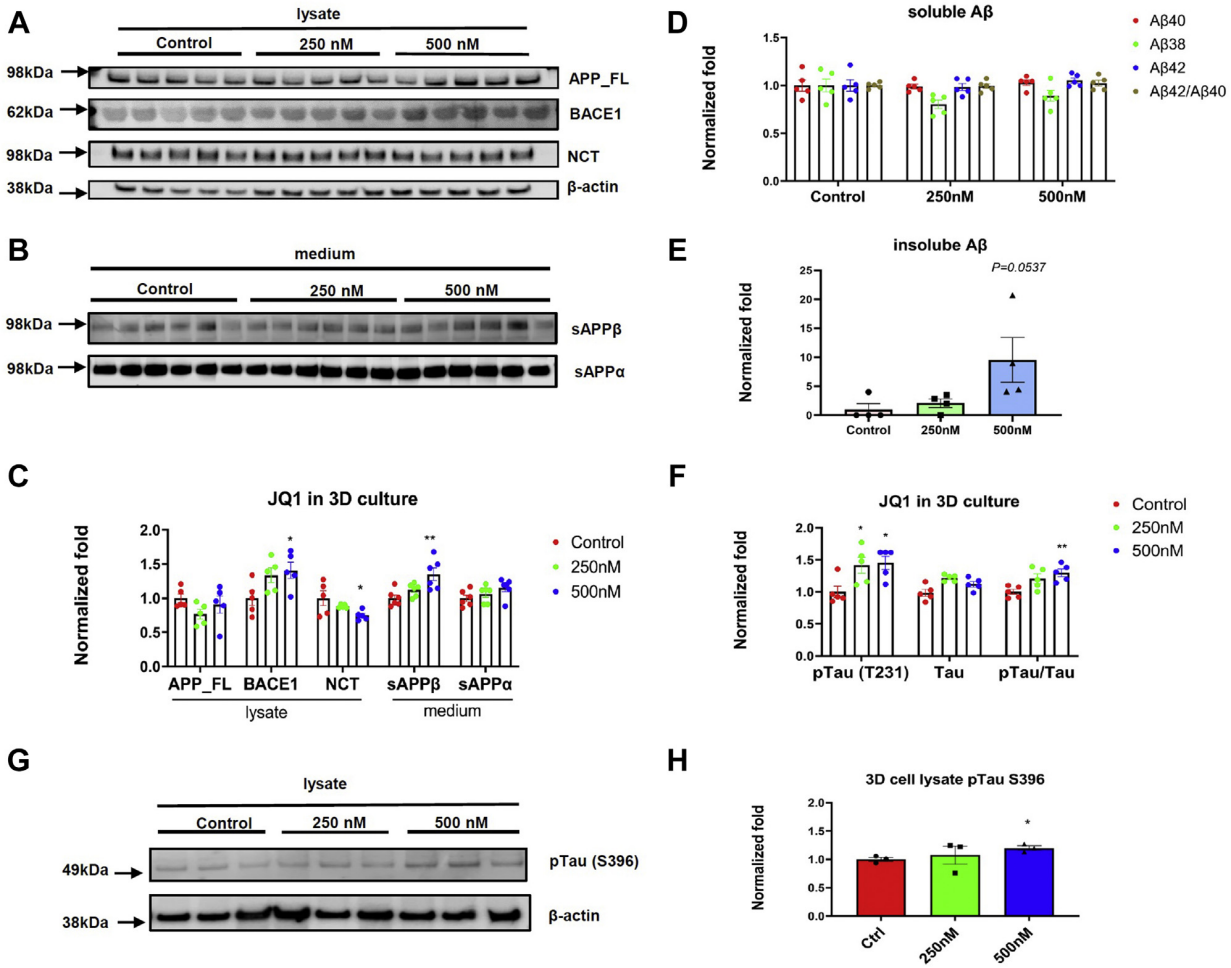
**Analysis of pathological Tau proteins by JQ1 in 3D-AD human neural cell culture model.** The 3D-AD human neural culture cells were differentiated for 5 weeks and then treated with JQ1 (250 nM and 500 nM) or vehicle (DMSO) for 2 weeks and applied to AD-related protein analysis, including WB (*A*–*C*), MSD-Aβ (*D*–*E*) and MSD-Tau for total and p-Tau (Thr231)(*F*) and WB for p-Tau (Ser396) (*G*–*H*). *A*–*C*, WB analysis for APP (by G12A antibody), BACE1, NCT and β-actin (as a control protein) in cell lysates and sAPPα/β proteins in medium. n = 5 to 6. *D*, MSD-Aβ analysis for soluble Aβ levels in medium. n = 5. *E*, MSD-Aβ analysis for insoluble Aβ42 from the matrix-gel extraction. n = 4. *F*, MSD-Tau analysis for Tau-/p-Tau (Thr231) levels in cell lysates. n = 4. *G*–*H*, WB analysis for p-Tau (Ser396) levels in cell lysates. The antibodies for p-Tau (Ser396) and β-actin (as a control protein) were utilized. n = 3. Mean ± SEM; ∗/∗∗/∗∗∗∗ showed significance; ∗*p* < 0.05; ∗∗*p* < 0.01; ∗∗∗∗, *p* < 0.0001. NCT, nicastrin.

**Supplemental Figure S4:**
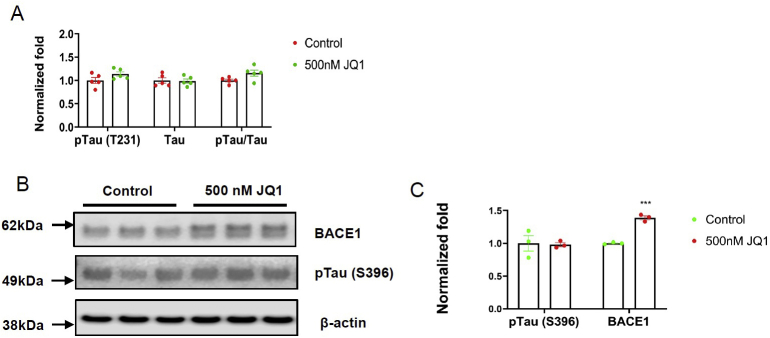


Next, we utilized our previously reported 3D-AD human neural culture model (7) to study AD-related Tau protein changes. Particularly the human neural progenitor cells expressing the *APP* Swedish and London mutations and the *PSEN1* δE9 mutation were seeded and 3D-differentiated to neurons and astrocytes. These cells were treated with 250 nM and 500 nM JQ1 or vehicle (DMSO) for 2 weeks. The lysates and medium were used by WB to detect APP expression and processing pathway. We found that 500 nM JQ1 increased levels of BACE1 and sAPPβ and decreased NCT level comparing with control (*p* < 0.05), without significantly changing APP-FL levels (*p* > 0.05) (Fig. 5, *A*–*C*). Additionally, MSD-Aβ analysis was performed to measure soluble Aβ levels from medium and insoluble Aβ levels from lysates extracted by formic acid. We showed that 250 nM or 500 nM JQ1 did not significantly affect soluble Aβ levels in the conditioned medium (Fig. 5*D*). A dose-dependent increase of insoluble Aβ42 levels in lysates was observed in cells treated with 250 and 500 nM JQ1 compared with vehicle (Fig. 5*E*). The *p* value of 500 nM JQ1 arrived at 0.0537 (Fig. 5*E*). Furthermore, the lysates were applied to MSD-Tau analysis, which enabled the detection of both total Tau and phosphorylated Tau (T231). We showed that 250 nM and 500 nM JQ1 significantly increased pTau levels compared with control (*p* < 0.05). For example, the 250 nM JQ1 was related to an increase up to 141.6% ± 27.9% (*p* < 0.05; *versus.* control). In total, 250 nM and 500 nM JQ1 led to dose-dependent increases of pTau/Tau ratios, with 500 nM JQ1 associated with an increase up to 129.8% ± 13.1% (*p* < 0.05; *versus.* control) (Fig. 5*F*). Additionally, we performed WB to detect pTau (S396) and found that 500 nM JQ1 moderately but significantly led to an increase of pTau (S396) levels (*p* < 0.05; *versus.* control) (Fig. 5, *G* and *H*). Because Tau phosphorylation in our 3D-AD culture model is dependent on FAD mutation and amyloid pathology (7), we next studied the effects of JQ1 on Tau phosphorylation in naïve 3D human neural culture that does not contain FAD mutations. This will allow us to test whether effects of JQ1 on tau phosphorylation are through Aβ-independent mechanism. Our MSD-Tau analysis on cell lysates showed that 500 nM JQ1 did not significantly change pTau (T231) levels in naïve 3D culture (*p* > 0.05) (Fig. S4*A*). Our WB analysis for pTau (S396) also showed an absence of significant change in pTau (S396) levels as a function of JQ1 (Fig. S4, *B* and *C*). Thus, these findings suggest that JQ1 affects tau phosphorylation using an amyloid pathology-dependent mechanism due to FAD mutations in our 3D-AD human neural culture model. Furthermore, consistent with H4 and our 3D-AD model cells, we found that JQ1 significantly increased BACE1 levels in naïve 3D human neural culture model (Fig. S4, *B* and *C*). Collectively, these results showed JQ1-related increase of not only AD-related Aβ, but also pathological Tau proteins, which will be useful to evaluate if JQ1 and molecules in its class can be a potential therapeutic for AD.

### Analysis of autophagy-related LC3 levels after ARV-825 and JQ1 treatment

**Supplemental Figure S5:**
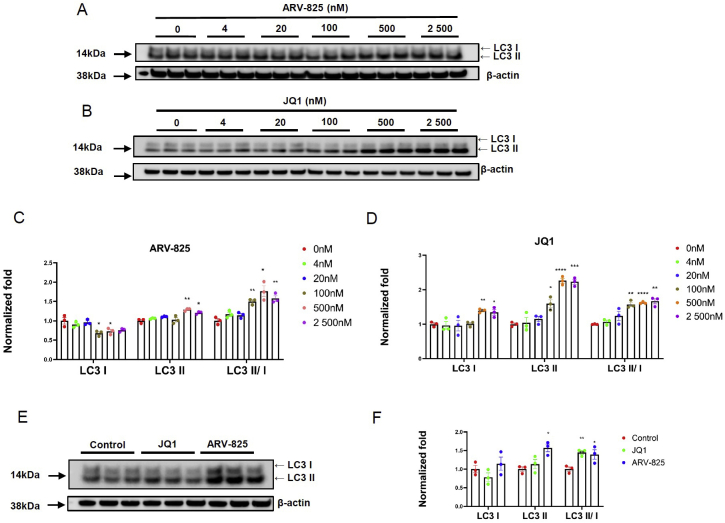


Previous results showed that downregulation of BRD4 can activate autophagy (17). Therefore, we next studied if downregulation of BRD4 by ARV-825 and JQ1 changes autophagy. Because studies also showed that autophagic activity may involve in the degradation of BACE1(43), we reasoned that ARV-825 or JQ1 may regulate BACE1 and Aβ levels by changing autophagy. Specifically, we investigated if ARV-825 (nM; 4, 20, 100, 500, and 2500) and JQ1 (nM; 4, 20, 100, 500, and 2500) may affect autophagy-related LC3A/B protein changes in H4-APP751. The cells were treated for 18 h and then harvested, with the cell lysates subsequently applied to WB to analyze autophagy using the LC3A/B antibody. We observed that ARV-825 (Fig. S5, *A* and *C*) and JQ1 (Fig. S5, *B* and *D*) dose-dependently increased LC3A/B-II:I ratios, suggesting an upregulation of autophagy in H4-APP751 cells, as expected and consistent with previous results (17). We also conducted analysis using JQ1 or ARV-825 on H4 naïve cells. The H4 naïve cells were treated with 100 nM ARV-825 or 500 nM JQ1 for 18 h and then harvested and analyzed by WB. We showed that JQ1 and ARV-825 moderately but significantly increased LC3A/B-II:I ratios compared with control (Fig. S5, *E* and *F*). Thus, our results suggested a mechanism of upregulating BACE1 and Aβ levels independent of autophagy, while our results agreed with previous report showing that BRD4 downregulation is related to autophagy activation (17).

## Discussion

Our current study evaluated the effects and translational potentials of targeting an epigenetic component, BRD4, on AD using cell models of AD. There has been keen interest in targeting BRD4 in clinical trials, which has led to many compounds such as BET inhibitors and PROTACs developed for trials in this area. JQ1 and OTX015 (original component of ARV-825) are both highly selective inhibitors of BET protein, that primarily target BRD4 (28, 35). Recently, preclinical evidence showed BRD4 with promising translational potentials. Thus, we performed studies by either inhibition of BRD4 by JQ1 or degradation of BRD4 by ARV-825. The significance and innovation of our study are discussed in the following aspects.

First, our results showed significant effects of both degradation and inhibition of BRD4 on Aβ generation and APP processing in cell models. The amyloid hypothesis of AD supports the cellular pathology of AD characterized by Aβ increase and subsequent Tau phosphorylation (1, 7). Aβ generation, or the amyloidogenic event, occurs through the APP proteolytic processing, which involves β-secretase and γ-secretase (43, 44). This pathway starts with β-secretase-mediated APP cleavage, followed by γ-secretase cleavage to produce Aβ. In addition to BRD4-related Aβ increase, we utilized the 3D human neural cell model of AD to study AD-related tau changes (7). We showed an upregulation of increased insoluble Aβ42, Tau protein phosphorylation, and increased BACE1 levels as a function of BRD4 inhibition in our 3D-AD human neural culture model. Collectively, our results supported the amyloid hypothesis and suggested an association of BRD4 downregulation with exacerbated amyloid and tau pathology in AD cell models.

Next, regarding the clinical potential of targeting BRD4 for AD, our results in this study did not support such an application in AD. JQ1 has been previously investigated to determine its potential as an AD therapeutic. Our current study has provided new insights in this domain. While JQ1 improves brain plasticity with positive effects on cognitive performance in wild-type mice and rescues cognitive deficits in AD transgenic APP/PS1 mice (34), it shows negative effects on neuronal activity and behavorial tests in wild-type mice in a different study (22). The discrepancy of these findings might be explained by the employed behavorial testing paradigm in these two studies. Furthermore, Magistri and colleagues showed that JQ1 reduces pTau (S396) levels in the brain of 3xTg AD mice overexpressing APP/PS1/Tau (30). Different from these animal-based findings, our current study characterized AD-related changes, particularly Aβ levels and APP processing in H4 cells as well as Tau phosphorylation, in our 3D-AD human neural culture model. Particularly, we found increased Tau phosphorylation for both pTau (S396) protein and pTau (T231) protein in our 3D-AD neural culture cells. We note that our pTau findings differed from those by Magistri and colleagues, which may be due to various models and mutations expressed in these models. While we used 3D-AD human neural culture model expressing FAD mutations in APP and PSEN1 without expressing Tau mutations, the 3xTg AD mice contained Tau P301L, a Tau mutation that was not implicated in AD. Additionally, not only did we show an increase of Aβ generation, but we also showed increased BACE1 levels in cells treated with ARV-825 or JQ1. Changes of BACE1 and BACE1-mediated APP processing have been extensively investigated for mechanisms in association with AD (45). Previous studies showed that an increase in BACE1 elicits neurodegeneration in animal models (46) and may trigger mitochondrial defects and mitophagy (47). Furthermore, BACE1 has important physiological roles, one of which is related to cleavage of the insulin receptor and neuronal BACE1 knock-in induces systemic diabetes in mice (48, 49). Although not suitable for AD, BRD4 has been considered in other conditions. Because BRD4 binding with P-TEFb is vital to expression of key G1 and growth-associated genes, the downregulation of BRD4 will lead to G1 cell cycle arrest and apoptosis (13). BET inhibition is considered a potential therapy in cancers and has also been implicated in other diseases such as type-I diabetes and antifibrosis in animal models of heart failure (23, 24, 25, 26, 27).

Furthermore, our study has provided new knowledge of BRD4 on AD-related β- and γ-secretase. This is the first study, to our knowledge, which describes β- and γ-secretase levels can be regulated by epigenetic regulation of bromodomain protein in association with APP processing. Previous studies showed that autophagy activation is a mechanism that reduces BACE1 levels by promoting its turnover (50). We showed an upregulation of autophagy as a function of BRD4 downregulation by JQ1 and ARV-825, which associated with increased BACE1 levels. We also showed that the mechanism of BACE1 increase was on a posttranscriptional level because our RT-qPCR results showed that *BACE1* mRNA levels did not change as a function of BRD4 downregulation. Furthermore, we showed that Aβ(42:40) ratios were not significantly changed by BRD4 downregulation, nor the levels of PS1-CTF. Interestingly, we found that BRD4 downregulation by both JQ1 and ARV-825 decreased NCT levels, suggesting BRD4 as a molecule that may endogenously enhance the expression of NCT. Thus, the collective data of our current study and previous findings suggest complex mechanisms underlying BRD4-medidated regulation of the neuropathological events of AD.

Notably, we showed a posttranscriptional mechanism by which JQ1 and ARV-825-mediated inhibition or degradation of BRD4 increases Aβ levels through regulating BACE1 and APP processing in AD cell models. A study by Benito *et al* (34) was focused on identifying candidate genes differentially expressed by JQ1 in both AD transgenic and wild-type animals. The authors found that the transgenes encoding for PS1 and APP were not differentially expressed in JQ1-treated AD transgenic APP/PS1-21 mice (34). Furthermore, the genes that were significantly differentially expressed in wild-type mice as a function of JQ1 did not include genes encoding proteins involving APP processing and amyloidogenesis (APP, BACE1, and γ-secretase) (34). These results further support a posttranscriptional mechanism of JQ1-related increase of BACE1 and APP proteins, consistent with our cell-based findings. It was possible that BRD4 may target candidate genes other than *BACE1* or *APP* on transcriptional levels, which then subsequently affect BACE1 and APP levels. The pathways/genes by which BRD4 affects BACE1 and APP processing in our AD model cells may associate with BRD4-related cell cycle and proliferation (CDKN1B, NRF2) or autophagy (18, 51, 52), which warrants future studies. Interestingly, using an oxidative stress-dependent activity, BRD4 binds and regulates the transcriptional factors p53 and SP1 (53, 54), which have been related to the transcription of APP and BACE1 in AD (55, 56). These results showed complex circuits for the regulatory role of BRD4 in transcriptional gene regulation. It is consistent with previous findings showing that BRD4 displays both enhancer and repressor functions for transcriptional regulation of target gene expression (52) with effects dependent on pathophysiological conditions (22, 34).

**Supplemental Figure S6:**
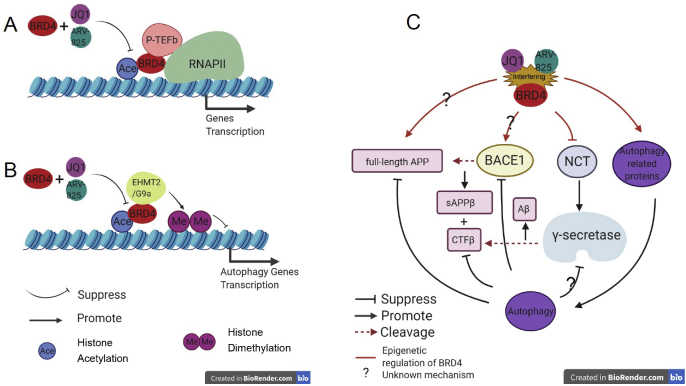


Finally, the results of our current study and previous studies support a representative model, which shows that JQ1/ARV-825-mediated inhibition or degradation of BET/BRD4 increases amyloidogenesis in AD (Fig. S6). Previous findings showed that BRD4 interacts with P-TEFb and RNAPII resulting in transcription upregulation of genes for cell growth and neurodevelopment (13, 14, 15). The small-molecule-based inhibitor JQ1 competitively binds the bromodomains of BRD4 and displaces BRD4 from accessing acetylated histones. Thus, the inhibition or degradation of BRD4, due to respectively JQ1 and ARV-825, may reduce BRD4 interaction with these partners and subsequently affect the downstream gene and protein expression. Furthermore, previous evidence also showed that targeting BRD4 affects transcriptional regulation of autophagy and lysosome genes (17). Particularly, reduced BRD4 binding with the promoters and the histone methyltransferase EHMT2/G9a, which represses expression of autophagy genes by dimethylating (MeMe) H3K9, may activate transcription of these genes (17, 18). Here, we provided evidence that JQ1/ARV-825 raises Aβ levels using a mechanism by which increases BACE1 protein levels through a posttranscriptional level. Furthermore, BACE1 and γ-secretase-related NCT displayed opposite direction of change, with NCT reduced as a function of BRD4 downregulation by ARV-825/JQ1 (Fig. S6). Our results demonstrated complex mechanisms in regulation of BRD4 and provided new insights in targeting BRD4 toward drug development.

In summary, our study has provided novel and translational insights for targeting BRD4 in AD clinical applications. We showed that pharmacological degradation and inhibition of BRD4 significantly increased Aβ levels that are related to AD neuropathology in cell models. Thus, regarding clinical potential, our results suggest that downregulation of BRD4 may not be a good therapeutic strategy for AD.

## Experimental procedures

### Reagents and antibodies

Chemicals, including ARV-825 and JQ1, were from Medchemexpress (Catalog #s: HY-16954 and HY-13030, respectively) and their stocks were in DMSO. The G12A antibody was used to detect APP full length and C-terminal fragments (APP-CTFs) (rabbit polyclonal, clone C7 targeting amino acid residues 732–751 of APP751, custom made by Thermo Fisher Scientific) and were previously reported (57, 58, 59). The sAPPβ antibody was purchased from IBL (Catalog #: 18975). The 6E10 antibody (BioLegend) is a monoclonal antibody (mAb) reactive to amino acid residues 1–16 of Aβ from the N-terminal sequence and can detect sAPPα in medium. The nicastrin (NCT) antibody (Catalog #: 5665), BACE1 antibody (Catalog #: 5606), and LC3 a/b antibody (Catalog #: 12741) were purchased from Cell Signaling. The BRD4 antibody was from BETHYL (Catalog #: A700-004-T). The β-actin antibody was from Sigma and used as a protein control. The antibody for pTau (S396) was from Cell Signaling (Catalog #: 9632). The HRP-conjugated secondary antibodies (anti-mouse and anti-rabbit) were from Pierce. The dilution factors for primary and secondary antibodies were 1: 1000 and 1: 10,000, respectively.

### Cell culture

The H4 human neuroglioma cells stably expressing 751-amino acid isoform of human APP (H4-APP751 cells) have been previously described (60, 61). The cells were cultured and maintained in conditioned medium (CM) that consists of Dulbecco’s modified Eagle's medium (DMEM) supplemented with 10% fetal bovine serum, 2 mM L-glutamine, 100 units per ml penicillin, 100 μg per ml streptomycin, and 200 μg per ml G418. Cells were cultured at 37 °C in a water-saturated air/5% CO_2_ atmosphere. The H4 naïve cells were cultured in the medium without G418. Cells were treated, and the conditioned medium and lysates were collected and applied to downstream analyses.

The three-dimensional (3D) human neural culture and treatment were carried out using the previously published protocol (7, 62). Briefly, the HReN-mGAP cells expressing APP Swedish and London mutations and the PSEN1 δE9 mutation (3D-AD) and the naïve 3D cells without expressing FAD mutations were utilized. The cells were cultured and plated at a cell density of 2.0 × 10^7^ cells per ml in a mixture of Matrigel and then 3D-differentiated for 5 weeks, followed by treatment for an additional 2 weeks. The conditioned medium and lysates were collected and analyzed.

### Cell lysis and protein amount quantification

Cell conditioned medium and lysates in regular tissue cultures were prepared using previously reported methods (60, 63). Briefly, cells were lysed in M-PER (Mammalian Protein Extraction Reagent, Thermo Fisher Scientific) containing 1× Halt protease inhibitor cocktail (Thermo Fisher Scientific). The lysates were collected and centrifuged at 10,000*g* at 4 °C for 20 min. The pellets were then discarded, and the supernatants were transferred to a new Eppendorf tube. Total protein was quantified using the BCA protein assay kit (Pierce). For 3D tissue culture, the cells were lysed with Lysis Buffer 6 (R&D Systems), the lysates were centrifuged at 10,000*g* for 20 min. The supernatants were utilized for MSD-Tau analysis. The pellets were used to detect insoluble Aβ levels by further extraction with 70% formic acid and centrifugation at 50,000*g* at 4 °C for 1 h. The supernatants were collected, neutralized with 1 M Tris base buffer, and applied to Aβ-MSD analysis.

### Western blotting (WB) analysis

WB analysis was carried out by the method previously described (58, 60, 63). Briefly, protein samples were extracted and applied to electrophoresis using the Novex NuPAGE SDS-PAGE Gel System (Thermo Fisher Scientific), followed by membrane transfer, antibody incubation, and signal development. The house-keeping protein, β-actin, was used as an internal control. The Odyssey Fc with Image Studio imaging software (LI-COR) was used to develop the blots and quantify the proteins of interest according to protocols previously described (58, 60, 63).

### AlamarBlue analysis

The alamarBlue assay was used to assess cell viability and proliferation rate and has been reported previously (61). In brief, the alamarBlue agent (Invitrogen; Catalog #: DAL1025) was added to cell culture medium at a final concentration of 10% (v/v), medium was collected after 4 h of incubation, and the fluorescence intensity was read using a 550-nm excitation and a 590-nm emission wavelength. The readouts from the different experimental groups were compared with those from the control group.

### Aβ and Tau protein measurements

Aβ and Tau measurements were performed following the reported protocols (59, 62, 64). In brief, proteins, including the 4G8 MSD-Aβ triplex (Catalog #: K15199E-2; Aβ40, Aβ38, and Aβ42) and tau (Catalog #: K15121D-2; total and phosphorylated tau/Thr231) were quantified using the multiarray electrochemiluminescence-based MSD assay. Briefly, the 96-well plates were first blocked with manufacture-provided diluent with shaking for 1 h at room temperature. Next, the samples and the protein standards were prepared and incubated on a shaker overnight at 4 ^°^C, followed by washing and then adding the reading buffer. Subsequently, the electrochemiluminescence signals were captured by the MesoScale SQ120 system. Protein levels of the samples were analyzed based on the protein standards. Protein levels from the treatment groups were compared and normalized to those from the control group.

### Immunofluorescence (IF) staining

The H4-APP751 cells were cultured and maintained in four-chamber culture slides as described above, then treated with DMSO, 500 nM JQ1, or 100 nM ARV-825 overnight. The slides were fixed with 4% paraformaldehyde (PFA) for 20 min at room temperature (RT), then permeabilized and blocked by incubating with a blocking PBS solution 0.1% Triton-X-100, containing 5% donkey serum, at RT for 1 h. After washing with PBS washing buffer containing 0.1% (v/v) Tween-20, cells were incubated with primary antibody 6E10 (anti-APP antibody, 1:250 dilution) and BACE1 (D10E5 from CST, 1:250) in the blocking solution at 4 °C overnight. After washing three times with washing buffer, the cells were then incubated with AlexaFluor secondary antibody (Alexa Fluor 488 anti-mouse, Catalog # A-21202; Alexa Fluor 546 anti-rabbit, Catalog # A10040, Life Technologies) for 2 h at RT. After washing five times, the cells were incubated with DAPI in PBS solution to stain the cell nucleus at RT for 10 min. The cells were then washed with PBS three times. To avoid fluorescence quenching, a drop of anti-fade gold (Life Technologies; Catalog # P36934) was added on top of the fixed/stained cells before imaging. The fluorescence images were captured using the Olympus DSU confocal microscope (Olympus USA).

### RNA extraction and quantitative RT-qPCR

Isolation of total RNA was performed using the RNeasy Mini kit (Qiagen) following previously reported methods (60) and manufacturer’s suggested protocols. RNA concentrations were measured using a NanoDrop ONE spectrophotometer (Labtech International). RNA (1 μg) was reverse transcribed using Oligo(dT)_20_ and SuperScript III First-Strand Synthesis System (Invitrogen). RT-qPCR reactions were carried out in a 10-μL reaction volume containing 500 nM of each primer and PowerUp SYBR Green Master Mix (Applied Biosystems) and 5 ng cDNA. The RT-qPCR amplification conditions were set up as follows: 2 min at 50 °C, 2 min at 95 °C, followed by 40 cycles of 15 s at 95 °C, 1 min at 60 °C according to the manufacturer suggested protocol, on a CFX96 Touch Real-Time PCR Detection System (BIO-RAD). The PCR cycle number that generated the first fluorescence signal above a threshold (threshold cycle, CT; 10 SDs above the mean fluorescence generated during the baseline cycles) was determined, and a comparative CT method was then used to measure relative gene expression (65). Primer sequences for APP, BACE1, and NCT were previously reported (66, 67, 68) and those for β-actin were from SuperScript III First-Strand Synthesis System (Invitrogen) (Table S2).

### Statistical analysis

The Student’s two-tailed *t* test was applied for statistical analysis comparing two groups. The one-way ANOVA analysis followed by Dunnett’s test was used for analysis of more than two groups in comparison to the control group. The results were presented as mean ± SEM. *p* < 0.05 was considered to be significant. Statistical analysis was performed using the PRISM GraphPad software.

## Data availability

All data are contained within the article.

## Supporting information

This article contains supporting information (13, 14, 15, 17).

## Conflict of interest

The authors declare that they have no conflict of interest with the contents of this article.

## Figures and Tables

**Supplemental Table S1:**
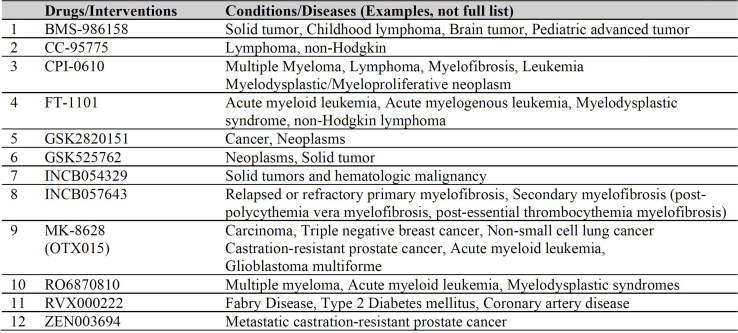


**Supplemental Table S2:**